# Novel Molecular Consortia of Cannabidiol with Nonsteroidal Anti-Inflammatory Drugs Inhibit Emerging Coronaviruses’ Entry

**DOI:** 10.3390/pathogens12070951

**Published:** 2023-07-18

**Authors:** Anna Pawełczyk, Rafał Nowak, Monika Gazecka, Anna Jelińska, Lucjusz Zaprutko, Paweł Zmora

**Affiliations:** 1 Department of Organic Chemistry, Pharmaceutical Faculty, Poznan University of Medical Sciences, 60-780 Poznan, Poland; zaprutko@ump.edu.pl; 2 Department of Molecular Virology, Institute of Bioorganic Chemistry, Polish Academy of Sciences, 61-704 Poznan, Poland; rnowak@ibch.poznan.pl (R.N.); mgazecka@ibch.poznan.pl (M.G.); 3 Department of Pharmaceutical Chemistry, Poznan University of Medical Sciences, 60-780 Poznan, Poland; ajelinsk@ump.edu.pl

**Keywords:** cannabidiol (CBD), nonsteroidal anti-inflammatory drugs (NSAIDs), molecular consortia, SARS-CoV-2, virus entry

## Abstract

The COVID-19 pandemic provoked a global health crisis and highlighted the need for new therapeutic strategies. In this study, we explore the potential of the molecular consortia of cannabidiol (CBD) and non-steroidal anti-inflammatory drugs (NSAIDs) as novel antiviral dual-target agents against SARS-CoV-2/COVID-19. CBD is a natural compound with a wide range of therapeutic activities, including antiviral and anti-inflammatory properties, while NSAIDs are commonly used to mitigate the symptoms of viral infections. Chemical modifications of CBD with NSAIDs were performed to obtain dual-target agents with enhanced activity against SARS-CoV-2. The synthesised compounds were characterised using spectroscopic techniques. The biological activity of three molecular consortia (CBD–ibuprofen, CBD–ketoprofen, and CBD–naproxen) was evaluated in cell lines transduced with vesicular stomatitis virus-based pseudotypes bearing the SARS-CoV-1 or SARS-CoV-2 spike proteins or infected with influenza virus A/Puerto Rico/8/34. The results showed that some CBD–NSAID molecular consortia have superior antiviral activity against SARS-CoV-1 and SARS-CoV-2, but not against the influenza A virus. This may suggest a potential therapeutic role for these compounds in the treatment of emerging coronavirus infections. Further studies are needed to investigate the efficacy of these compounds in vivo, and their potential use in clinical settings. Our findings provide a promising new approach to combatting current and future viral emergencies.

## 1. Introduction

The search for completely new chemical entities is an established tendency in modern medical chemistry, particularly in the era of coronaviruses and various multifactorial diseases. Recent research has shown that the following guidelines are extremely important in the design of new, relevant therapeutic structures:

- the design of new chemical systems based on innovative structural features [1], such as hybrids and conjugates (molecular consortia in general);
- the use of compounds of natural origin, i.e., phytochemicals, with pleiotropic activity, which have been effective in the treatment of many diseases [2];
- the use of small-molecule drugs and their new derivatives, with known and proven therapeutic power [3].

These studies were based on both natural and small-molecule chemical individuals, such as natural and small-molecule cannabidiol (CBD) and selected small-molecule nonsteroidal anti-inflammatory drug (NSAID) compounds, and used the synthetic trends mentioned above to create new chemical entities with potential biological activity. CBD is the main non-psychoactive constituent of *Cannabis sativa,* and has exhibited a wide range of therapeutically promising pharmacological effects, either as a single drug or in combination with other drugs in adjunctive therapy. CBD is one of the chemically and phytogenetically related phenolic terpenes derived from natural hemp. The chemistry and pharmacology of CBD, as well as various molecular targets including CBD receptors and other CBD-interactive components of the endocannabinoid system, have been reviewed extensively [4,5,6,7]. The preliminary results of many studies have prompted the exploration of the therapeutic potential of CBD in relation to various diseases, particularly cancer and drug-resistant epilepsy [7,8,9,10,11,12].

Recently, CBD has become famous due to reports of its health-promoting properties in the treatment of COVID-19. Research results have indicated that CBD can help regulate oxygen levels in the body and protect against cytokine storms. It is speculated that CBD may suppress the immune system’s reaction because CBD resembles endocannabinoids naturally occurring in the human body [13]. Recent research results have shown that high concentrations of CBD have a beneficial effect on the treatment of acute respiratory distress syndrome caused by COVID-19, restoring proper oxygen levels in the body [14]. In addition, laboratory tests have shown that CBD reduces swelling and scarring in the lungs. In summary, CBD can help reduce cytokine levels, thus improving the level of oxygen in the body and supporting the regeneration of damaged lung tissue. Therefore, CBD can help fight against the dangerous effects of a coronavirus infection [13,14]. It has also been established that CBD-rich extracts can help regulate the expression and/or activity of the SARS-CoV-2 cellular receptor (i.e., ACE2). Studies have shown that 13 extracts potent in CBD, which were extremely low in psychoactive substances, effectively modulated ACE2 expression [15].

The concepts of structural hybridisation and molecular consortia are well established and have led to the approval of numerous antiviral and anticancer drugs [1,16]. However, it is very important to skilfully select the active agents that will be covalently combined to obtain dual-target activity. NSAIDs are analgesic, antipyretic and anti-inflammatory medications widely used to relieve symptoms related to many conditions, including viral disorders [16,17]. Their use is often limited due to side effects, particularly gastrointestinal ulcerogenic activity and renal toxicity. Therefore, introducing another pharmacophore to mask the free carboxylic group is a promising approach to designing dual-target agents. For example, nitric oxide-releasing NSAIDs, such as NO-aspirin and an ibuprofen derivative, which contain a cleavable ester linker to a nitric oxide-releasing moiety, have been reported to have a superior anti-inflammatory and antithrombotic profile [18]. It has also been suggested that CBD and its physical mixtures with selected NSAIDs (ibuprofen and diclofenac) are promising candidates for the adjuvant treatment of high-risk vulvar squamous cell carcinoma patients [19]. Regarding the side effects of NSAIDs, various combinations with CBD may become a new therapeutic solution. The results of studies on the use of CDB and NSAIDs, as well as on the combined treatment of CBD together with NSAIDs, provide the foundation for a new approach to the modern treatment of many diseases.

Therefore, the aim of this study was to analyse the antiviral effect of selected novel molecular consortia consisting of CBD and NSAID units.

## 2. Materials and Methods

### 2.1. General Procedure for the Synthesis of CBD-NSAID Molecular Consortia

The CBD was purchased from Medcolcanna Organics Inc. (Calgary, AB, Canada), and NSAIDs, i.e., ibuprofen, ketoprofen and naproxen, as well as dicyclohexylcarbodiimide (DCC), 4-(dimethylamino)pyridine (DMAP), and solvents (dichloromethane, hexane, ethyl acetate), were purchased from Aldrich^®^ (Sigma Aldrich, Schnelldorf, Germany), Fluka^®^ (Buchs, Switzerland), POL-AURA (Warsaw, Poland), Chempur^®^ (Piekary Śląskie, Poland) and POCh^®^ (Gliwice, Poland).

**Figure 1:**
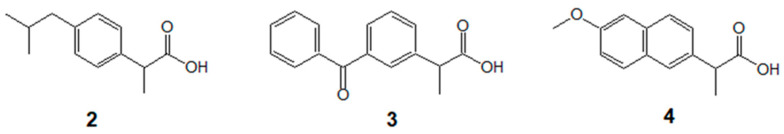
NSAID structures selected (**2**—ibuprofen; **3**—ketoprofen; **4**—naproxen).

In a 50 mL flask, CBD (**1**) (0.314 g, 1 mmol) and selected NSAID compounds (Figure 1; **2–4**) (2.2 mmol) were dissolved in dry CH_2_Cl_2_ (10 mL). 

**Figure 2:**
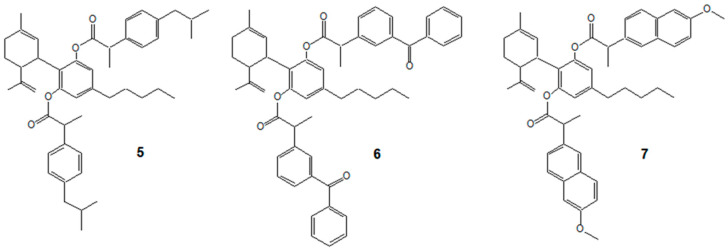
Chemical formulas of CBD-NSAID molecular consortia (**5**–**7**) obtained.

**Scheme 1:**
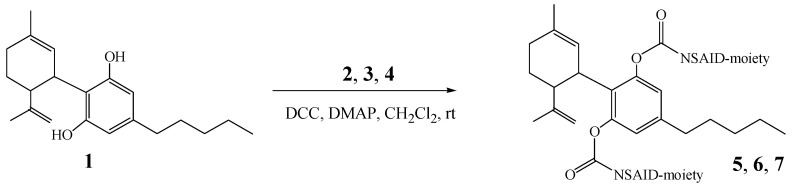
General synthetic pathway of CBD-NSAID molecular consortia (**1**—CBD, **2**—ibuprofen, **3**—ketoprofen, **4**—naproxen, **5**—CBD-ibuprofen, **6**—CBD-ketoprofen, **7**—CBD-naproxen).

Then, DCC (0.454 g, 2.2 mmol) and DMAP (0.012 g, 0.1 mmol) were added (Figure 2). The reaction mixture was stirred at ambient temperature until the reaction was completed (about 2 h). After this time, the mixture was filtered (from the resulting dicyclohexylurea), washed with 1N aqueous HCl solution, saturated brine solution and dried over anhydrous MgSO_4_. The obtained mixture was concentrated to yield crude product (**5–7**) as a white waxy solid. The new hybrid diester was purified with flash column chromatography on silica gel using a hexane/ethyl acetate (9:1 or 9:2, v:v) mixture as eluent. The synthetic route designed and employed to obtain the proposed hybrid derivatives **5**–**7** is presented in Scheme 1. All the derivatives were prepared according to the previously reported method [20,21].

The ^1^H- and ^13^C-NMR spectra were recorded using a BRUKER spectrometer, 400 and 100 MHz, respectively (AvanceCore Select, Bruker, Billerica, MA, USA). Chemical shifts were expressed in parts per million (ppm), relative to tetramethylsilane (TMS) as an internal standard, using CDCl_3_ as a solvent. MS spectra were recorded on a 402 AMD INTECTRA apparatus (AMD Intectra GmbH, Harpstedt, Germany) using the electron impact technique (EI), operating at 75 eV. The IR spectra were recorded using an ATR-FTIR spectrometer Nicolet iS50 (Thermo Scientific, Waltham, MA, USA), and UV-VIS spectra were recorded using an LLG-uniSPEC 2 spectrophotometer (Lab Logistics Group GmbH, Meckenheim, Germany). The progress of the reactions and the purity of products were checked using the thin-layer chromatography (TLC) method on silica gel plates (DC-Alufolien Kieselgel 60 F_254_ from Merck, Darmstadt, Germany). A mixture of hexan/ethyl acetate (9:1, 9:2, 9:4, *v*/*v*) was used as the eluent. The TLC spots on the plates were observed in UV light (λ = 254 nm). Silica gel 60 (63–200 μm particle size, Merck) was used for column chromatography.

In details, the CBD-ibuprofen molecular consortium (**5**; CBD-I) was synthesised via the reaction of 0.314 g of **1** and 0.454 g of **2**, and after chromatographic separation, the product **5** was obtained as a colourless resin (0.559 g, 81%), R_f_ = 0.55 (hexane/ethyl acetate 9:1).

^1^H NMR (δ[ppm]): 7.10–7.03 (4H, m, H_Ar-I_), 6.79–6.95 (m, 4H, H_Ar-I_), 6.34 (s, 2H, H_Ar-CBD_), 4.97 (d, 1H, C=CH, *J* = 10.4), 4.26–4.09 (m, 2H, C=CH_2_), 4.09 (s, 1H, CH), 4.00 (s, 1H, CH), 3.15–2.85 (d, 2H, CH, *J* = 10.4), 2.32 (d, 4H, CH_2_, *J* = 6.8), 2.30–2.27 (m, 6H, CH_3_), 1.95–1.79 (m, 2H, CH), 1.82–1.67 (m, 6H, CH_3_), 1.49–1.34 (m, 4H, CH_2_), 1.23–1.02 (m, 8H, CH_2_), 0.75–0.69 (m, 15H, 5 × CH_3_).

^13^C NMR (δ[ppm]): 2.73 (C=O_ester_), 148.02, 147.88, 147.73, 141.82, 137.46, 136.86, 132.10, 129.54, 129.43, 127.39, 127.14, 124.51, 110.68, 60.68, 45.44, 45.07, 38.08, 37.63, 35.19, 31.53, 30.23, 28.97, 23.21, 22.41, 22.38, 19.54, 18.64, 14.04.

EI-MS (*m*/*z*): 691.5 [M]^+^ (C_47_H_62_O_4_), 501.3, 434.1, 313.1, 231.1, 187.9, 160.9, 118.9.

FTIR (ATR, cm^−1^): 1756.14 (C=O_ester_)

The CBD-ketoprofen molecular consortium (**6**; CBD-K) was produced via the reaction of 0.314 g of **1** and 0.559 g of **3**; after chromatographic separation, the product **6** was obtained as a colourless resin (0.448 g, 57%), R_f_ = 0.62 (hexane/ethyl acetate 9:2).

^1^H NMR (δ[ppm]): 7.69–7.59 (m, 8H, H_Ar-K_), 7.50–7.44 (m, 4H, H_Ar-K_), 7.37–7.31 (m, 6H, H_Ar-K_), 6.42 (m, 2H, H_Ar-CBD_), 5.00 (d, 1H, C=CH, *J* = 10.4), 4.96 (s, 2H, C=CH_2_), 4.27–4.20 (m, 2H, CH), 3.19–3.17 (d, 1H, CH, *J* = 10.4), 2.87–2.84 (d, 1H, CH, *J* = 10.4), 2.39–2.29 (m, 6H, CH_3_), 1.95–1.75 (m, 6H, CH_3_), 1.54–1.35 (m, 4H, CH_2_), 1.24–1.05 (m, 8H, CH_2_), 0.76–0.70 (m, 3H, CH_3_).

^13^C NMR (δ[ppm]): 196.38 (C=O), 172.11 (C=O_ester_), 147.58, 142.11, 140.50, 139.96, 138.13, 137.99, 137.42, 137.57, 131.74, 130.08, 130.03, 129.51, 129.27, 128.88, 128.37, 126.00, 125.90, 124.32, 124.25, 110.85, 45.69, 45.54, 45.44, 45.23, 35.22, 31.61, 31.53, 30.61, 30.49, 28.94, 23.42, 22.69, 22.44, 19.49, 19.32, 14.16, 14.04.

EI-MS (*m*/*z*): 787.0 [M]^+^ (C_53_H_54_O_6_), 549.6, 467.1, 313.2, 230.4, 209.1, 104.8.

FTIR (ATR, cm^−1^): 1755.61 (C=O_ester_), 1656.81 (C=O).

The CBD-naproxen molecular consortium (**7**; CBD-N) was obtained via the reaction of 0.314 g of **1** and 0.506 g of **4**; after chromatographic separation, the product **7** was obtained as a colourless resin (0.480 g, 65%); R_f_ = 0.57 (hexane/ethyl acetate 9:2).

^1^H NMR (δ[ppm]): 7.62–7.58 (m, 6H, H_Ar-N_), 7.37–7.34 (d, 2H, H_Ar-N_), 7.00–7.04 (m, 4H, H_Ar-N_), 6.40 (s, 2H, H_Ar-CBD_), 5.02 (s, 1H, C=CH), 4.26 (s, 1H, C=CH_2_), 4.19 (s, 1H, C=CH_2_,), 3.81 (s, 6H, OCH_3_), 3.10–3.08 (d, 2H, CH, *J* = 10.4), 2.37–2.28 (m, 2H, CH), 1.94 (s, 3H, CH_3_), 1.75–1.73 (d, 3H, CH_3_-C=C, *J* = 10.4), 1.59–1.57 (d, 6H, CH_3_, *J* = 6.8), 1.38–1.34 (m, 4H, CH_2_), 1.18–1.12 (m, 8H, CH_2_), 0.79–0.70 (m, 3H, CH_3_).

^13^C NMR (δ[ppm]): 172.63 (C=O_ester_), 157.69, 147.73, 141.93, 134.68, 133.85, 132.18, 129.33, 128.96, 127.27, 126.24, 125.94, 124.37, 119.03, 110.83, 105.51, 55.34, 45.58, 45.43, 37.95, 35.16, 31.62, 31.51, 30.41, 28.88, 23.19, 22.23, 19.23, 14.17, 14.03.

EI-MS (*m*/*z*): 738.7 [M]^+^ (C_49_H_54_O_6_), 525.5, 418.4, 313.4, 169.6.

FTIR (ATR, cm^−1^): 1751.25 (C=O_ester_).

### 2.2. Cells and Viruses

The HEK293T (CRL-3216, ATCC), MDCK.2 (CRL-2936, ATCC), A549 (CCL-185, ATCC), BHK-21 (G43) (a kind gift of Prof. Gert Zimmer, Institute of Virology and Immunology, Bern, Switzerland, [20]), and Vero-TMPRSS2 (a kind gift of Prof. Stefan Pöhlmann, German Primate Center—Leibniz Institute for Primate Research, Göttingen, Germany, [21]) were cultivated in Dulbecco’s modified Eagle’s medium (DMEM, Gibco, Carlsbad, CA, USA) supplemented with 10% foetal bovine serum (FBS, Gibco) and 100 U/mL penicillin and streptomycin (P/S, Gibco). Every third/fourth passage, BHK-21 (G43) and Vero-TMPRSS2 were additionally supplemented with 0.5 μg/mL hygromycin B and 100 μg/mL zeocin, and 50 μg/mL blasticidin, respectively. I1-hybridoma cells (CRL-2700, ATCC) were maintained in minimal essential medium (MEM, Gibco) supplemented with 15% FBS and 100 U/mL P/S. The influenza virus A/Puerto Rico/8/34 (IAV PR8) was reconstituted from the previously described 8-plasmid system [22] and propagated on the MDCK.2 cells. The IAV PR8 viral titre was measured with quantitative RT-PCR, as previously described [23].

### 2.3. The Cytotoxicity Analysis of CBD-NSAID Molecular Consortia

The HEK293T, A549, and Vero-TMPRSS2 were seeded in 96-well plates at a density of 2 × 10^5^ cell/mL and incubated overnight at 37 °C and 5% CO_2_. Next, the medium was removed, cells were washed with warm PBS, and then a medium containing serial dilutions of CBD-NSAID molecular consortia at a final concentration of 1, 5, 10, 50 and 100 μg/mL or mock-treated medium was added. After 24 h incubation at 37 °C, cytotoxicity was analysed with a CellTiter-Glo 2.0 Cell Viability Assay (Promega, Madison, WI, USA), according to manufacturer’s instructions. Briefly, the culture medium was carefully removed and 50 μL/well of the reagent was added. After 10–15 min of incubation at room temperature, the lysates were transferred to the white 96-well plates, and the luminescence was checked with Hidex Sense Plate Reader.

### 2.4. CBD-NSAID-Mediated Inhibition of SARS-CoVs Virus Entry

To investigate the CBD-NSAIDs’ effect on the SARS-CoVs virus entry, we used the previously described [24] surrogate system based on VSV*ΔG-FLuc pseudotypes bearing SARS-CoV-1 and SARS-CoV-2 S proteins, as well as VSV g protein as a control. The VSV*ΔG-FLuc pseudotypes encoding GFP and firefly luciferase were propagated on transgenic BHK-21 (G43) cells (a kind gift of Prof. Gert Zimmer, Institute of Virology and Immunology, Mittelhäusern and Bern, Bern, Switzerland), which express the VSV g protein after mifepristone induction. Next, to produce VSV-pseudotypes bearing SARS-CoVs S proteins, the harvested VSV-pseudotypes were used to transduce the HEK293T cells previously transfected with plasmids encoding SARS-CoV-1 and SARS-CoV-2 S proteins, i.e., pCAGGS-SARS-CoV-1-S and pCG1-SARS-2-S (a kind gift of Prof. Stefan Pöhlmann, German Primate Center—Leibniz Institute for Primate Research, Göttingen, Germany, [21]). After 1 h incubation of transfected HEK293T cells with VSV-pseudotypes, the supernatant was removed, cells were gently washed with warm PBS, and fresh culture medium containing I1-hybridoma cell-produced anti-VSV g antibodies was added. The supernatants containing VSV-SARS-CoVs-S pseudotypes were collected at 48 h post transduction.

To examine the effect of the CBD-NSAID molecular consortia on the virus entry, the VSV-SARS-CoVs-S pseudotypes were incubated for 1 h at 37 °C with CBD-NSAIDs at a final concentration of 1, 5, 10, 50 and 100 μg/mL or mock-treated. After incubation, the CBD-NSAID-pretreated pseudotypes were added to HEK293T cells transfected with previously described [21] plasmids encoding ACE-2 and TMPRSS2, and left for 1 h at 37 °C. Next, the cells were gently washed with warm PBS and fresh culture medium was added. The luminescence was investigated at 24 h post transduction with ONE-Glo substrate (Promega) on a Hidex Sense plate reader.

### 2.5. The CBD-NSAID Molecular Consortia’s Effect on Influenza A Virus Replication

To find the CBD-NSAID molecular consortia’s mechanisms of action, we analysed their effect on the IAV replication cycle. For this, we seeded A549 and Vero-TMPRSS2 cells in 24-well plates at a density of 2 × 10^5^ cells/mL. After 24 h, IAV PR8 was incubated for 1 h at 37 °C with CBD-NSAIDs at a final concentration of 1, 5, 10, 50 and 100 μg/mL, or mock-treated. Next, the cells were gently washed with warm PBS and then infected with CBD-NSAID-pretreated IAV PR8 at an MOI of 0.01. After 1 h incubation, the infection medium (DMEM supplemented with 0.3% BSA) containing IAV PR8 was removed, cells were washed with PBS, and a fresh infection medium with CBD-NSAID molecular consortia at a final concentration of 1, 5, 10, 50 and 100 μg/mL or mock-treated was added. Additionally, the fresh infection medium for the A549 cells was supplemented with the 2.5 μg/mL N-acetylated trypsin (Sigma). Culture supernatants were collected at 48 h post infection, and the number of viral RNA genome copies were determined by RT-qPCR, as described previously [23]. Briefly, the viral RNA was isolated from cell-free supernatants with a NucleoSpin RNA Virus Kit (Macherey Nagel, Oensingen, Switzerland) according to the manufacturer’s protocol. For determination of the amount of viral RNA, the RT-qPCR was carried out using a previously described IAV gene-specific primer and probe [23], and a One Step RT-qPCR kit (Takara, Kusatsu City, Japan). The RT-qPCR protocol consists of 20 min incubation at 50 °C, 15 min denaturation at 95 °C, 40 cycles of denaturation at 94 °C for 15 s, and annealing with extension at 60 °C for 60 s. The reaction was performed on a BIO-RAD CFX96 Real-Time system. To define the limit of detection (LOD) and to generate a standard curve to calculate the absolute copy number from the cycle threshold (Ct) value, the specific standards were made as 10-fold dilutions of RNA transcribed on an IAV PR8 template. Six serial dilutions starting from 3.6 × 10^7^ the total number of viral RNA genome copies were used. In addition, we checked the IAV structural proteins’ expression in the infected cells after the 100 μg/mL CBD-NSAID treatment with an immunofluorescence microscope. Briefly, the cells were infected with 100 μg/mL CBD-NSAID-pretreated IAV PR8 at an MOI or mock-treated. At 48 h post infection, the cells were fixed and stained with anti-IAV antibodies and corresponding secondary antibodies conjugated with FITC, and DAPI. Moreover, we analysed the IAV protein expression on the virion after CBD-NSAID treatment. The IAV was incubated with 100 μg/mL CBD-NSAIDs for one hour, concentrated on sucrose cushion, denatured at 95 °C for 5 min, and then transferred on the nitrocellulose membrane and stained with polyclonal anti-IAV antibodies (Merck, Germany) and corresponding anti-goat antibody coupled with FITC (Merck, Germany).

### 2.6. Statistical Analysis

Data are expressed as mean *±* SD. The IC_50_ was calculated using a nonlinear regression analysis. Statistical analyses were performed with GraphPad Prism.

## 3. Results

### 3.1. NSAID-CBD Molecular Consortia Synthesis

Inspired by the latest literature reports, small-molecule structures of natural CBD and already proven anti-inflammatory NSAIDs have been chemically combined into a single molecule termed the CBD–NSAID molecular consortium. Molecular hybridisation of two or more bioactive molecules via a covalent bond is an effective and efficient tool for the development of new drug candidates. Regarding the importance of a medicinally interesting scaffold of CBD (**1**) and NSAIDs (**2**–**4**) (Figure 1), we decided to combine them into a single molecule, that is, a molecular consortium [1]. Three molecular consortia (**5**–**7**) consisting of CBD (**1**) and selected racemic NSAID structures, namely, ibuprofen (**2**), ketoprofen (**3**) and naproxen (**4**), were obtained (Figure 2).

CBD (**1**) was reacted with appropriate NSAID compounds (**2**–**4**) in the presence of DCC and DMAP reagents. DCC is an activating and coupling agent for carboxylic substrates, while strongly nucleophilic DMAP acts as an acyl transfer reagent in the Steglich esterification reaction [25,26], which is a highly useful process in the conversion of sterically demanding substrates. The water resulting from a DCC-mediated reaction reacts immediately with the carbodiimide applied, and forms dicyclohexylurea (DHU), which is insoluble in a reaction medium and precipitates as a white solid. Reactions were carried out in an anhydrous dichloromethane solution at room temperature for up to two hours to achieve full conversion of the CBD substrate.

As a result of the synthesis, three structures hybridised using the formulas shown in Figure 2 were obtained. Further isolation and purification via column chromatography resulted in the desired pure CBD–NSAID diesters **5**–**7** with yields ranging from 57% to 81%. The structure of the CBD–NSAID hybrid compounds was confirmed via ^1^H- and ^13^C-NMR, EI-MS, IR and UV-VIS data. In the IR spectrum, characteristic ester bands for CBD-I (1756.14 cm^−1^), CBD-K (1755.61 cm^−1^), CBD-N (1751.25 cm^−1^) are observed. In the case of the CBD-K derivative, an additional carbonyl band at 1656.81 cm^−^^1^ is present (Supplementary Figure S1). According to the spectrophotometric measurements, the main absorption maxima for CBD-I, CBD-K, and CBD-N occur in the ranges of 216–232 nm, 214–274 nm, and 214–248 nm, respectively (Supplementary Figure S2).

### 3.2. The Cytotoxicity of CBD-NSAID Molecular Consortia

**Figure 3:**
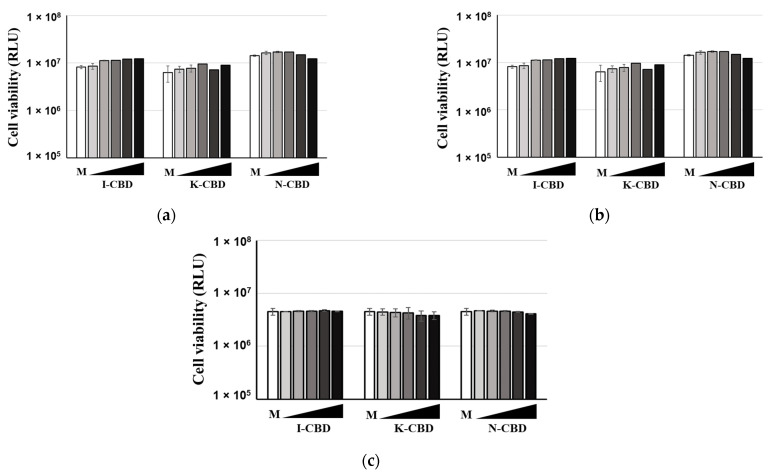
Cytotoxicity of the CBD-NSAID molecular consortia in HEK293T (**a**), Vero-TMPRSS2 (**b**), and A549 (**c**) cells. To analyze the cytotoxic effect of CBD-NSAIDs, the cells were treated with increasing concentrations of CBD-NSAIDs or were mock-treated (M, with an equal volume of the solvent DMSO). The cell viability was analysed at 24 h post CBD-NSAID treatment using the CellTiter-Glo Luminescent Cell Viability Assay Protocol (Promega). The results of representative experiments performed with triplicate samples are shown. Error bars indicate standard deviations (SD). Similar results were obtained in three independent experiments.

Three molecular consortia consisting of CBD and NSAIDs (i.e., ibuprofen, ketoprofen and naproxen) in a serial dilution of 0, 1, 5, 10, 50 and 100 μg/mL were tested for cytotoxic effect after 24 h incubation on HEK293T, A549, and Vero-TMPRSS2 cells. We did not observe any significant differences in cell viability between different CBD–NSAID concentrations and mock-treated cells (Figure 3), which suggests that the analysed molecular consortia did not have a cytotoxic effect.

### 3.3. The CBD-NSAIDs Effect on the SARS-CoVs Entry

**Figure 4:**
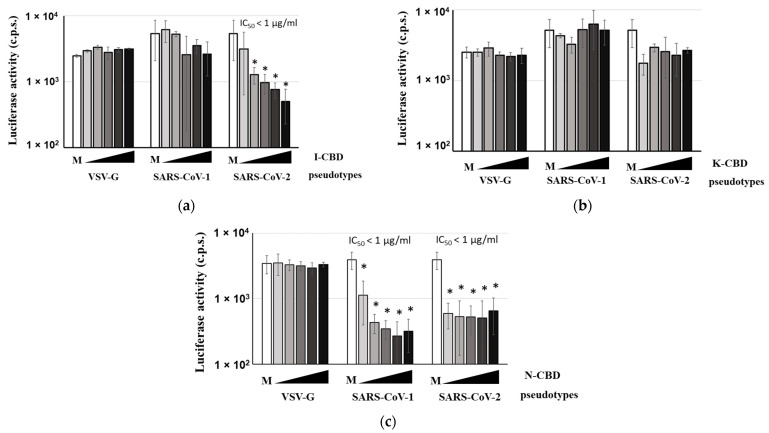
Inhibition of SARS-CoVs S protein-driven entry by the CBD-NSAID molecular consortia. The VSV-pseudotypes bearing VSV-G, SARS-CoV-1 or SARS-CoV-2 S proteins were incubated for 1 h with increasing concentrations of CBD-I (**a**), -K (**b**), -N (**c**), or were mock-treated (M), and then used for transduction of HEK293T cells expressing ACE-2 and TMPRSS2. Transduction efficiency was analysed at 24 h post inoculation by determining luciferase activities in cell lysates. The results of a single experiment carried out with triplicate samples are shown; error bars indicate standard deviations (SD). Similar results were obtained in three separate experiments. * *p* < 0.05.

To investigate the effect of CBD–NSAID molecular consortia on virus entry, we used the previously described VSV-pseudotype system encoding the reporter gene (i.e., luciferase). In this system, a decrease in luciferase activity indicates the inhibition of virus entry. As expected, no effect of CBD–NSAIDs was observed in cells transduced with VSV-pseudotypes bearing VSV g (Figure 4). A significant reduction in the SARS-CoV-1 virus entry was found when CBD-N was applied, with IC_50_ below 1 μg/mL. Moreover, the observed effect was clearly dose-dependent, with more than a 10-fold decrease in luciferase activity in transduced cells treated with 50 and 100 μg/mL, and an approximately 2-fold reduction when 1 μg/mL of CBD-N was applied. We did not find any significant inhibitory effect of CBD-I and CBD-K on SARS-CoV-1 virus entry (Figure 4). At the same time, CBD-I and CBD-N molecular consortia significantly inhibited SARS-CoV-2 entry, with an IC_50_ below 1 μg/mL. CBD-I reduced luciferase activity in SARS-CoV-2 transduced cells in a dose-dependent manner, with approximately 2- and 10-fold decreases when the lowest and highest concentrations were applied, respectively. A greater than 10-fold inhibition of SARS-CoV-2 entry was observed when CBD-N was added to the transduced cells. In this case, the lowest and highest concentrations had a similar inhibitory effect (Figure 4). The weakest inhibitory effect on SARS-CoV-2 entry was found in cells treated with CBD-K, with a non-significant decrease in luciferase activity for each concentration used.

### 3.4. The CBD-NSAIDs Effect on the IAV Replication

**Figure 5:**
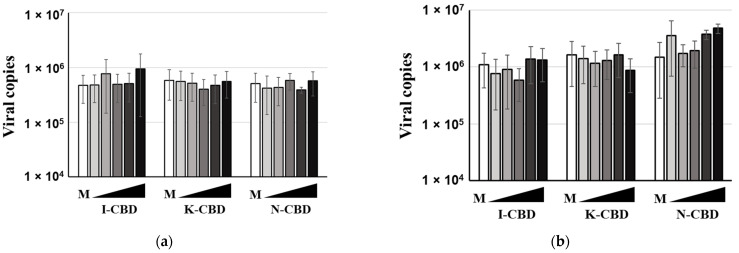
The effect of CBD-NSAID molecular consortia on influenza virus A/Puerto Rico/8/34 replication. The Vero-TMPRSS2 (**a**) and A549 (**b**) cells were infected with CBD-NSAID-pretreated IAV PR8 at an MOI 0.01, and then incubated with increasing concentrations of CBD-I, -K, or -N, or were mock-treated (M). At 48 h post-infection, viral spread was quantified as the release of infectious particles into the culture supernatants, as measured by an RT-qPCR. The result of a single experiment carried out with triplicate samples is shown; error bars indicate standard deviations (SD). Similar results were obtained in three separate experiments.

To analyse the effect of CBD–NSAID molecular consortia on influenza A virus replication, we infected A549 and Vero-TMPRSS2 with CBD–NSAID-pre-treated replication-competent IAV PR8, and then incubated the infected cells with increasing concentrations of CBD–NSAIDs. At 48 h post-infection, we analysed the viral copy numbers in the supernatants, and did not find any relevant differences between the mock and CBD–NSAID-treated groups (Figure 5). In addition, we found no significant differences in the IAV structural proteins’ expression in the infected cell line, i.e., almost all cells were infected with IAV, and no effect of CBD-NSAID molecular consortia treatment was observed (Supplementary Figure S3). Similarly, we did not observe any significant differences in the IAV structural proteins’ expression on the IAV virion level (Supplementary Figure S4). Because there were no differences in the IAV PR8 titres as well as IAV protein expression, we did not analyse immune responses, such as interferon signalling pathways and/or other host/viral gene expressions, any further.

## 4. Discussion

The ongoing COVID-19 pandemic has caused almost 700 million confirmed SARS-CoV-2 infections and approximately 7 million deaths (as of 20 April 2023) [27,28]. In addition to the development of safe and highly effective mRNA and vector vaccines against severe COVID-19 [29], finding new antiviral therapeutics remains a necessity. Non-vaccinated, not fully vaccinated and certain vaccinated individuals, such as older people, persons with comorbidities, and patients with immunodeficiencies, are high-risk groups for severe COVID-19, hospitalisation, and death [30,31]. Therefore, effective, globally available and relatively cheap COVID-19 treatment strategies, both those that inhibit virus replication and those that mitigate SARS-CoV-2-mediated immune system overreaction, are being investigated by scientists and pharmaceutical companies all over the world. Moreover, such strategies may be insensitive to acquired mutations in the SARS-CoV-2 genome, and thus to the emergence of new genetic variants with a different phenotype, which may result in higher transmissibility and virulence, as well as potential resistance to existing vaccines.

Currently, in the European Union, several drugs have been approved for the treatment of COVID-19: remdesivir (Gilead Sciences, Foster City, CA, USA), PF-07321332, and ritonavir (Pfizer, New York, NY, USA), and monoclonal antibodies, such as sotrovimab (GlaxoSmitkKline, Brentford, UK), casirivimab/imdevimab (Roche, Basel, Switzerland), regdanvimab (Celltrion, Yeonsu-gu, Republic of Korea), and tixagevimab/cilgavimab (AstraZeneca, Cambridge, UK) [32]. However, it should be highlighted that all the above-mentioned therapeutics (i) act directly against SARS-CoV-2 replication, i.e., remdesivir [33], PF-07321332, and ritonavir [34], or (ii) block virus entry to the host cells, i.e., monoclonal antibodies [35], and should be implemented as part of therapy within 1–5 days of the onset of symptoms to mitigate virus replication, spread, and pathogenesis, and to block the SARS-CoV-2-mediated overreaction of the immune system, which leads to severe acute respiratory syndrome. In addition, these drugs are recommended for SARS-CoV-2-infected patients who belong to the severe COVID-19 risk group in the first stages of viral infection [36]. Patients with a mild-to-moderate SARS-CoV-2 infection course are mainly treated symptomatically (e.g., via NSAID administration) [37,38,39]. The combination of direct antiviral compounds, such as CBD, with general anti-inflammatory drugs, such as ibuprofen, ketoprofen, and/or naproxen, may have beneficial effects on COVID-19 therapy in patients with mild and severe infection courses. At the same time, it should be noted that potential adverse drug events and drug–drug interactions with medical CBD may occur (i.e., simultaneous administration of CBD and naproxen affect CYP2C8/9), which may increase the risk of side effects related to the substrate; thus, monitoring for adverse effects and toxicity is needed [40]. Our study showed that the molecular consortia of CBD with NSAIDs did not have any cytotoxic effect in vitro, which may suggest that they are a safe alternative that does not impact the CYP450 enzymes implicated in the primary metabolism and biotransformation of most therapeutic agents and xenobiotics.

The antiviral activities of CBD have been confirmed by many scientific groups, which have also discussed potential antiviral mechanisms. Nguyen et al. observed that CBD and its metabolite 7-OH-CBD inhibit SARS-CoV-2 replication in lung epithelial cells by inhibiting viral gene expression in part via the upregulation of the host IRE1 alpha RNase endoplasmic reticulum stress response, as well as via interferon signalling pathways [41]. At the same time, Corpetti et al. demonstrated that CBD blocked SARS-CoV-2 S protein-induced cytotoxicity and inflammation through PPARγ-dependent suppression of proinflammatory agent signalling. In addition, the authors demonstrated that CBD inhibits S protein enterotoxicity by blocking the expression of interleukin 1 beta, interleukin 6, tumour necrosis factor alpha, and interleukin 18 [42]. Moreover, van Breemen et al. focused on the SARS-CoV-2 S protein as a target for CBD and its derivatives, namely cannabidiolic acid (CBDA) and cannabigerolic acid (CBGA). The authors demonstrated that CBDA and CBGA significantly block SARS-CoV-2 entry into cells via high affinity with the receptor-binding domain on the S1 subunit, which is responsible for the interaction with SARS-CoV-2 receptor ACE2 [43]. The following amino acids of the S1 subunit were essential for CBDA interactions: R403, Y453, Y495, G469 and Y505. It is worth mentioning that these amino acids are quite conserved and present in the S1 subunit of SARS-CoV-1 and SARS-CoV-2, as well as in almost all genetic variants of SARS-CoV-2 (i.e., alpha, beta, and delta), which means that CBD and its derivatives may be a universal anti-SARS-CoVs agent, independent of the emergence of new genetic variants. Additionally, our study showed that the molecular consortia CBD-I and CBD-N significantly reduced SARS-CoV-2 viral entry, measured as an inhibition of luciferase activity in a VSV bearing SARS-CoV-2 S protein pseudotypes. Furthermore, we observed that the analysed consortia were characterised by similar levels of IC_50_, as observed by Nguyen et al., and were significantly lower than those presented for CBDA and CBDG [41]. At the same time, the molecular consortium CBD-K did not significantly affect the entry of SARS-CoV-2 to the cells, which may be explained by the spatial hindrance caused by the two aromatic rings in its chemical structure. Interestingly, in the case of SARS-CoV-1, only the molecular consortium CBD-N exhibited significant inhibitory activity, with an IC_50_ below 1 μg/mL. This may be explained by the fact that the amino acids interacting with CBD are identical among the SARS-CoVs; however, in general, the S protein among the SARS-CoVs is 76% identical and 86% similar. The mutations in the receptor-binding sites of the S protein in the SARS-CoVs may result in different interactions with other proteins or small compounds with inhibitory activity. The use of linkers in the chemical structure of CBD–NSAID molecular consortia may potentially expand the specificity of the analysed compound to all emerging coronaviruses.

Our study had certain limitations. For example, the chosen cell culture model could not represent the inflammation process, and we could not check the anti-inflammatory activities of the CBD–NSAID molecular consortia. At the same time, at least one NSAID, (i.e., naproxen) exhibited broad anti-influenza virus activity by inhibiting nucleoprotein binding to RNA and impeding viral nucleoprotein nuclear export [44,45,46]. In our study, we did not find any effect of CBD-N on IAV replication, which means that the CBD-N molecular consortium does not interact directly with the IAV nucleoprotein, probably due to its size, but it may still have anti-inflammatory activity. The study of the anti-inflammatory activities of the synthesised CBD–NSAIDs should be continued using mice models.

## 5. Conclusions

Generation of bifunctional molecular consortia consisting of CBD and NSAIDs may result in a compound with higher antiviral activity. Simultaneously, it should be noted that some combinations of CBD and NSAIDs may result in the loss of their activity, due to the spatial hindrance and the impossibility of interaction with SARS-CoVs spike proteins. Therefore, the generated compounds should be carefully analysed with all available tools and models in the context of antiviral as well as anti-inflammatory activities. Further studies on the activity of CBD-NSAID molecular consortia should be continued with more complex models and in vivo models.

## Data Availability

The data presented in this study are available within this article.

## Supplementary Materials

The following supporting information can be downloaded at: https://www.mdpi.com/article/10.3390/pathogens12070951/s1, Figure S1: The IR spectra of synthesized CBD-NSAID molecular consortia; Figure S2: The UV-VIS spectra of synthesized CBD-NSAID molecular consortia; Figure S3: Effect of the CBD-NSAID molecular consortia on the influenza A virus (IAV) replication. The cells were infected with 100 μg/mL CBD-NSAIDs pretreated IAV PR8 at an MOI or mock treated. At 48 h post infection, the cells were fixed and stained with anti-IAV antibodies, corresponding secondary antibodies conjugated with FITC, and DAPI; Figure S4: Effect of the CBD-NSAID molecular consortia on the structural proteins of influenza A virus (IAV). The cells were infected with CBD-NSAIDs pretreated IAV PR8 at an MOI 0.01 and then incubated with 100 μg/mL CBD-I, -K, or -N or mock-treated. At 48 h post infection, the supernatant was collected and the IAV was concentrated on the sucrose cushion, denaturated at 95 °C for 5 min and then transfer on the nitrocellulose membrane. The, IAV proteins were stained with anti-IAV antibodies (Merck, Germany) and corresponding secondary antibodies coupled with horse radish peroxidase.
