# Supplementary material for: Novel Molecular Consortia of Cannabidiol with Nonsteroidal Anti-Inflammatory Drugs Inhibit Emerging Coronaviruses’ Entry
Source: Pathogens. 2023 Jul 18;12(7):951. doi: 10.3390/pathogens12070951 (PMC10383849; doi:10.3390/pathogens12070951)
Supplement: Supplementary file 1 [file pathogens-12-00951-s001.zip › pathogens-2433328-supplementary.pptx]

## Slide 1
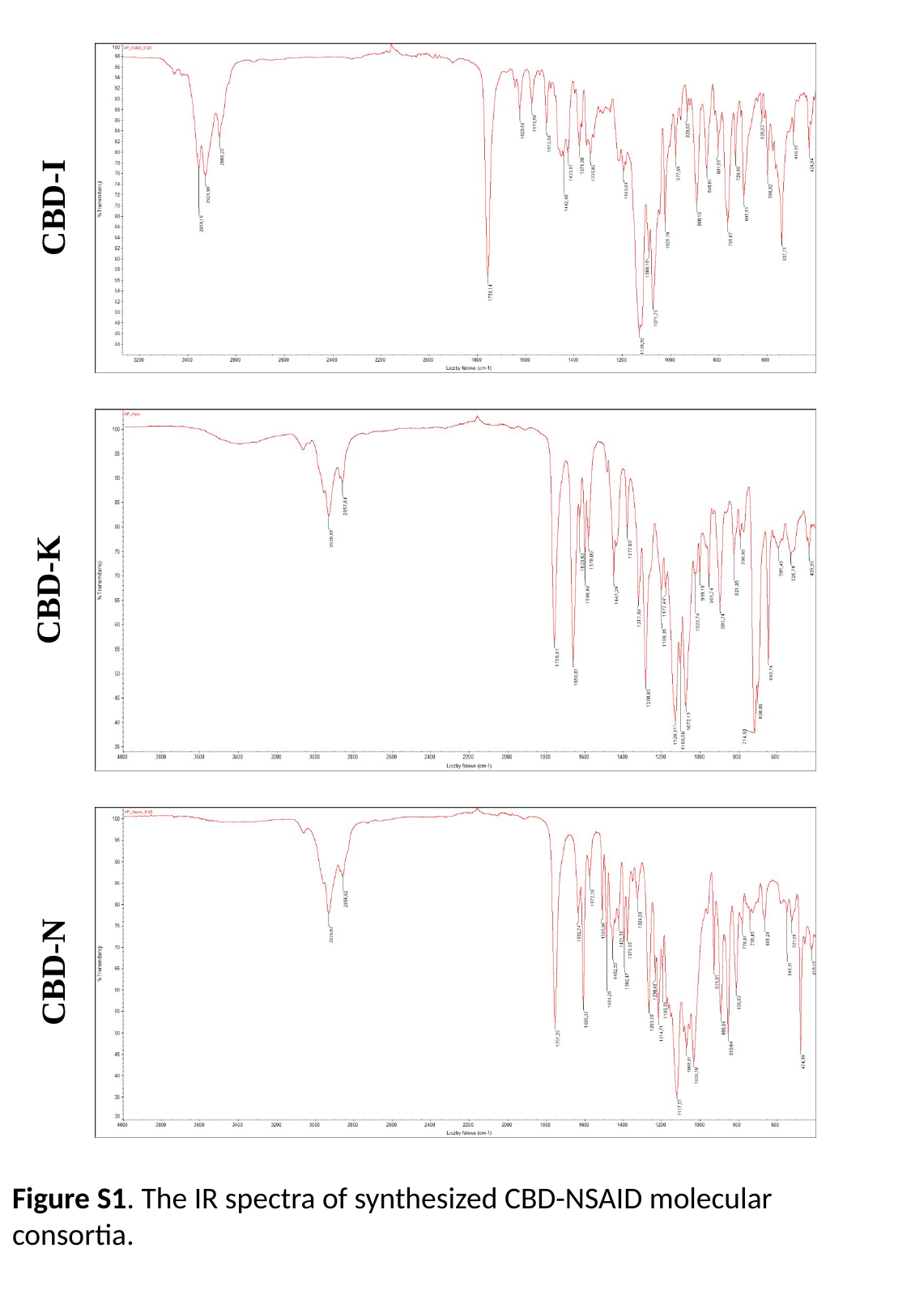

CBD-I
CBD-K
CBD-N
Figure S1. The IR spectra of synthesized CBD-NSAID molecular consortia.

## Slide 2
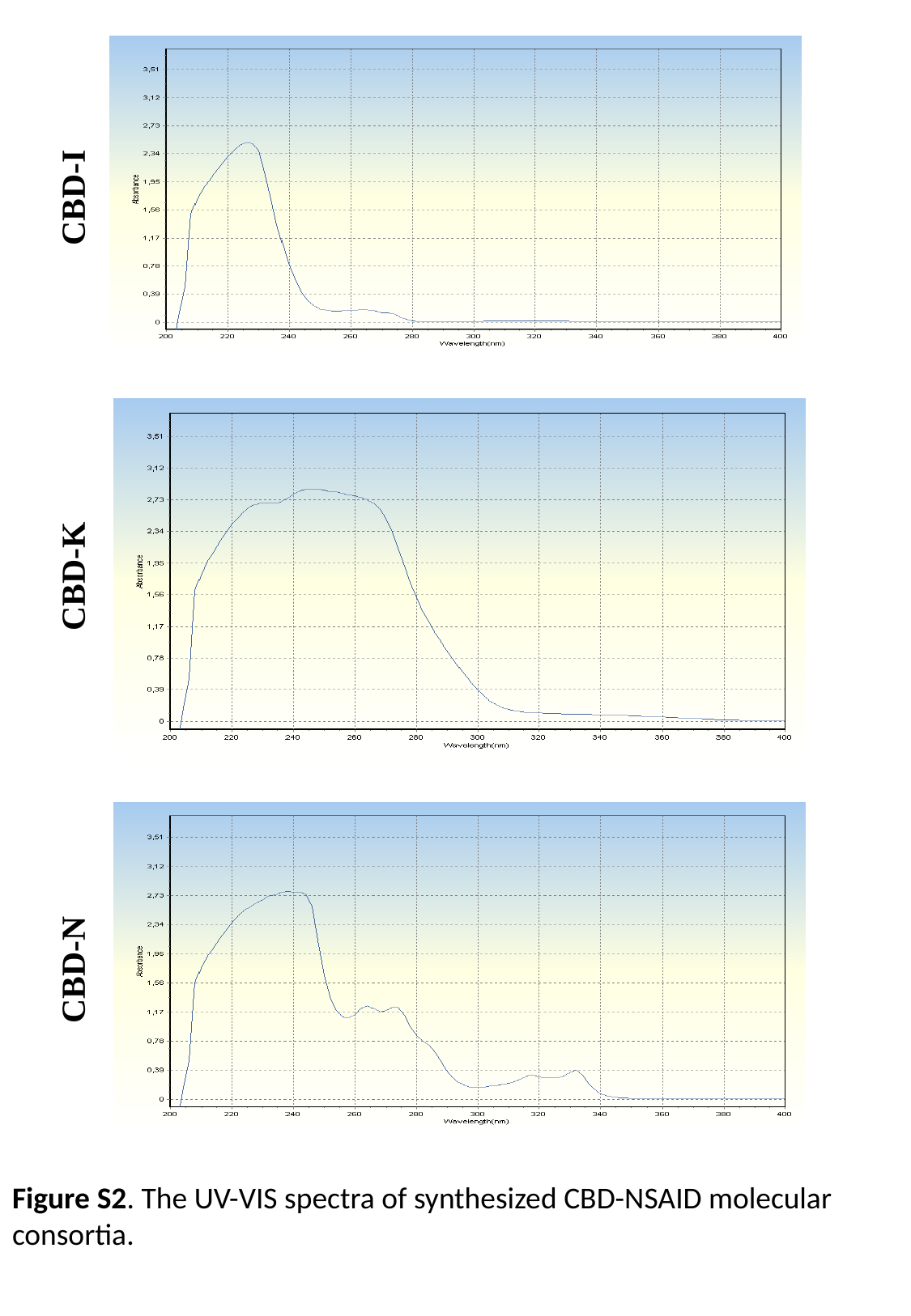

CBD-I
CBD-K
CBD-N
Figure S2. The UV-VIS spectra of synthesized CBD-NSAID molecular consortia.

## Slide 3
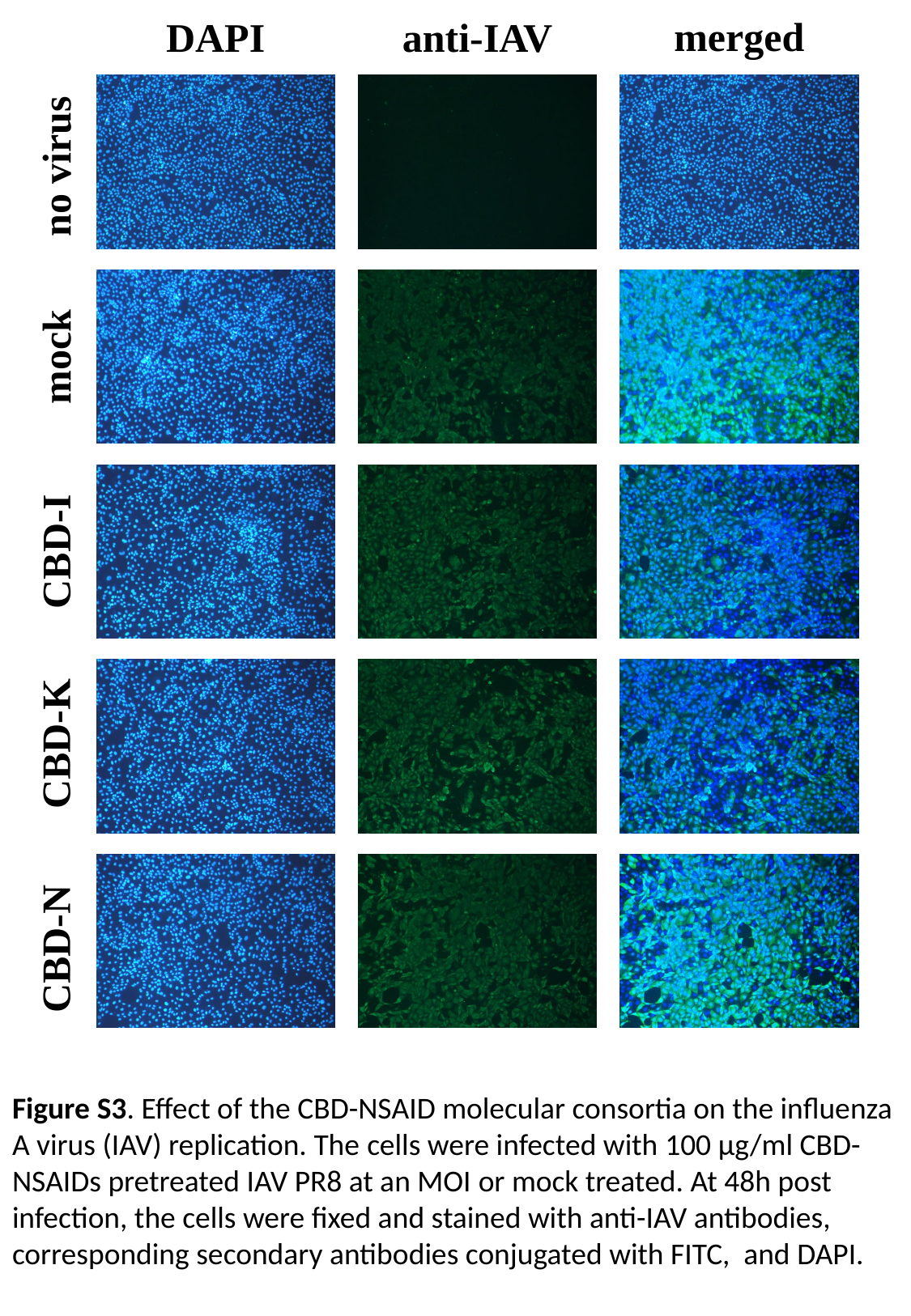

merged
anti-IAV
DAPI
no virus
mock
CBD-I
CBD-K
CBD-N
Figure S3. Effect of the CBD-NSAID molecular consortia on the influenza A virus (IAV) replication. The cells were infected with 100 μg/ml CBD-NSAIDs pretreated IAV PR8 at an MOI or mock treated. At 48h post infection, the cells were fixed and stained with anti-IAV antibodies, corresponding secondary antibodies conjugated with FITC, and DAPI.

## Slide 4
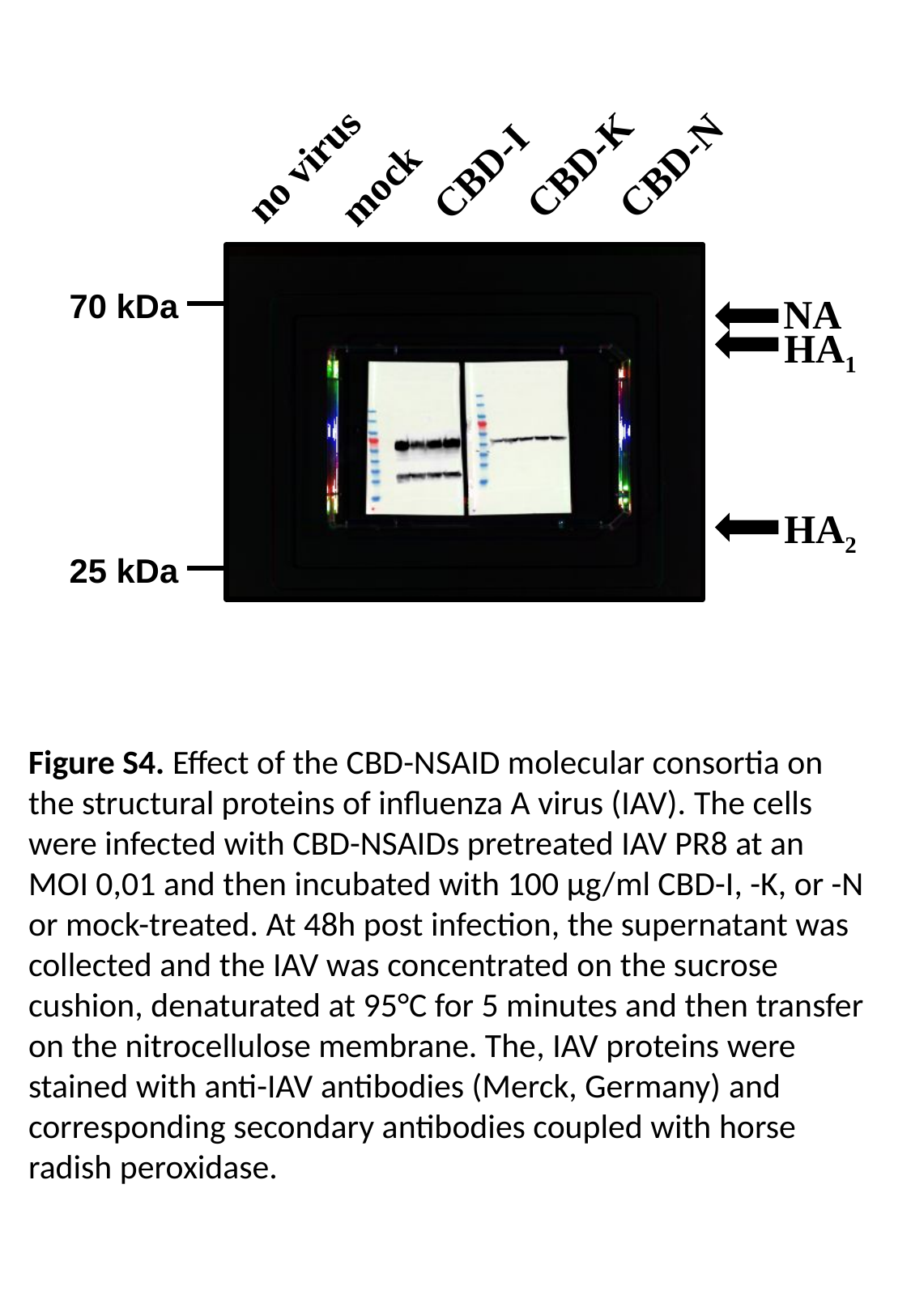

CBD-K
CBD-N
CBD-I
no virus
mock
70 kDa
NA
HA1
HA2
25 kDa
Figure S4. Effect of the CBD-NSAID molecular consortia on the structural proteins of influenza A virus (IAV). The cells were infected with CBD-NSAIDs pretreated IAV PR8 at an MOI 0,01 and then incubated with 100 μg/ml CBD-I, -K, or -N or mock-treated. At 48h post infection, the supernatant was collected and the IAV was concentrated on the sucrose cushion, denaturated at 95°C for 5 minutes and then transfer on the nitrocellulose membrane. The, IAV proteins were stained with anti-IAV antibodies (Merck, Germany) and corresponding secondary antibodies coupled with horse radish peroxidase.
